# Energetic and Neuromuscular Demands of Unresisted, Parachute- and Sled-Resisted Sprints in Youth Soccer Players: Differences Between Two Novel Determination Methods

**DOI:** 10.3390/s24227248

**Published:** 2024-11-13

**Authors:** Gabriele Grassadonia, Michele Bruni, Pedro E. Alcaraz, Tomás T. Freitas

**Affiliations:** 1 UCAM Research Center for High Performance Sport, UCAM Universidad Católica de Murcia, 30107 Murcia, Spain; palcaraz@ucam.edu (P.E.A.); tfreitas@ucam.edu (T.T.F.); 2 UPSS—International Department of Motor Arts, Popular University of Sport Sciences, 00122 Rome, Italy; 3 UPM—Department of Medical Sciences, Popular University of Milan, 20122 Milan, Italy; 4 MIU—Department of Sport Sciences, Miami International University, Miami, FL 33131, USA; 5 University eCampus, 220060 Novedrate, Italy; mik.bruni96@gmail.com; 6 Facultad de Deporte, UCAM Universidad Católica de Murcia, 30107 Murcia, Spain; 7 Strength and Conditioning Society, 30008 Murcia, Spain; 8 NAR—Nucleus of High Performance in Sport, São Paulo 04753-060, Brazil

**Keywords:** apparent efficiency, energy cost, metabolic power, speed loss, EMG, GPS-IMU

## Abstract

The aim of this study was to analyze the differences in terms of (1) muscle activation patterns; (2) metabolic power (MP) and energy cost (EC) estimated via two determination methods (i.e., the Global Positioning System [GPS] and electromyography-based [EMG]); and (3) the apparent efficiency (AE) of 30-m linear sprints in seventeen elite U17 male soccer players performed under different conditions (i.e., unloaded sprint [US], parachute sprint [PS], and four incremental sled loads [SS15, SS30, SS45, SS60, corresponding to 15, 30, 45 and 60 kg of additional mass]). In a single testing session, each participant executed six trials (one attempt per sprint type). The results indicated that increasing the sled loads led to a linear increase in the relative contribution of the quadriceps (R^2^ = 0.98) and gluteus (R^2^ = 0.94) and a linear decrease in hamstring recruitment (R^2^ = 0.99). The MP during the US was significantly different from SS15, SS30, SS45, and SS60, as determined by the GPS and EMG approaches *(p*-values ranging from 0.01 to 0.001). Regarding EC, significant differences were found among the US and all sled conditions (i.e., SS15, SS30, SS45, and SS60) using the GPS and EMG methods (all *p* ≤ 0.001). Moreover, MP and EC determined via GPS were significantly lower in all sled conditions when compared to EMG (all *p* ≤ 0.001). The AE was significantly higher for the US when compared to the loaded sprinting conditions (all *p* ≤ 0.001). In conclusion, muscle activation patterns, MP and EC, and AE changed as a function of load in sled-resisted sprinting. Furthermore, GPS-derived MP and EC seemed to underestimate the actual neuromuscular and metabolic demands imposed on youth soccer players compared to EMG.

## 1. Introduction

Achieving high acceleration rates and top speeds in sprinting efforts is a key factor in team-sports [1,2,3,4,5,6]. Therefore, training strategies to improve this type of action have been increasingly developed in recent years [7,8,9,10,11]. For example, loaded sled towing has been used as a common method for resistance sprint training [12,13,14,15], usually being prescribed based on a percentage of body mass or velocity loss (i.e., percentage of velocity loss relative to the maximum speed achieved) [11,16,17]. Sled towing has been found to increase ground contact time, trunk angle, hip flexion [11,18,19,20], and shoulder range of motion due to added resistance [3,4,19,20,21,22] in a load-dependent manner (i.e., the greater the load, the greater the kinematic changes). To replicate sprint mechanics with an additional load (i.e., as a secondary training method [8]), programs primarily use lighter loads, as heavier loads can lead to acute changes in running form and disrupt normal acceleration kinematics [3,4,11,23]. Therefore, appropriate load control helps achieve training specificity regarding movement patterns and speed [24,25,26]. When it comes to short to long-term adaptations to sled towing, this method has been shown to improve short-distance sprint performance (e.g., acceleration and transition phases) and change direction ability [27]. Sprint training without resistance, in turn, improves performance in the maximal speed phase; thus, it appears that each phase. Thus, it appears that each phase of sprint running requires a specific training approach [28,29,30,31,32,33].

Notably, most research on sprinting (loaded and unloaded) has predominantly focused on its neuromuscular determinants, while relatively little attention has been devoted to examining the energetic demands associated with these efforts. In this regard, di Prampero et al. [34] based on the study by Minetti et al. [35], estimated the energy cost (EC) and metabolic power (MP) of running using a biomechanical equivalence. This approach redefined ‘high-intensity’ and offered new possibilities for quantifying workload and assessing physical performance in training and competition. However, this method has yet to be applied in studies using sprints with resistance (e.g., sleds, parachutes, etc.) as the biomechanical and physiological differences from the original model may lead to discrepancies. Understanding the energetic demands of loaded sprinting could enable practitioners to optimize the application of this method by determining, for example, the most appropriate timing for its use (such as during specific phases of the competitive season or within a weekly microcycle). In this context, a recently proposed model [36] based on Colli [un-published], could be reapplied to address this issue and allow the study of the MP and EC of sprinting with an overload compared to that without external resistance.

Another parameter not studied in sprinting with resistance is apparent efficiency (AE). This metric corresponds to the ratio between total work and EC. The AE is based on various assumptions and tools [37,38,39,40,41], with the concept of estimating total mechanical work being controversial [1,21,22,41,42]. The sum of internal and external work represents the total work (mechanical and metabolic) performed to move the body over a given distance [42,43,44]. Therefore, previous studies have performed a kinematic-biomechanical analysis rather than a muscle-tendon analysis [38,40,41] and have not investigated AE during high-intensity actions such as loaded and unloaded sprinting. Considering that AE is derived from different modalities and movement patterns that are still unexplored, it is necessary to analyze this parameter during loaded and unloaded sprinting.

Hence, based on all of the above, the study aims to: (1) analyze the variations in muscle activation patterns (assessed as changes in relative contribution of the quadriceps, glutes and hamstring muscles) during sprints under different loading conditions; (2) investigate and compare the MP and EC of loaded and unloaded sprinting through the use of two novel methods (based on the Global Positioning System [GPS] and electromyography [EMG] measures) [36]; and (3) evaluate the AE of resisted sprint efforts. The hypotheses were that the relative muscle contribution would change as a load function, that MP and EC would be underestimated if determined using GPS technology, and that AE would be lower in resisted sprints compared to unloaded sprints. The novelty of this study lies in its comparative analysis of GPS and EMG technologies under varying resistance conditions, offering a dual-perspective approach that simultaneously captures both energetic and neuromuscular demands. This methodology addresses existing research gaps by providing a comprehensive understanding of load-induced adaptations during sprinting, thereby enhancing our knowledge of the physiological and biomechanical responses to loaded sprints in youth soccer players.

## 2. Materials and Methods

### 2.1. Study Design

A comparative cross-sectional study was designed and implemented with a convenience sample being recruited for the research. During the 2021/2022 preseason (i.e., July), data were collected from players of the same soccer club and category (under seventeen, U17) who were available to participate in the study. All measurements were performed during a single test session per player. The experimental procedures consisted of participants first completing four constant incremental runs of over 50 m to establish an ad hoc running profile [36]. Sprints over 30 m were then performed under different loading conditions, including an unloaded sprint (US), parachute sprint (PS), and sled sprints with loads of 15, 30, 45, and 60 kg (SS15, SS30, SS45, and SS60, respectively). During the weeks before the study, all participants were submitted, on multiple occasions, to parachute and sled sprinting stimuli to become familiarized with these specific methods. On the testing day, before data collection, a warm-up consisting of mobility and athletic running exercises was completed for approximately 15 min. The players were familiar with all the exercises suggested during the warm-up, as they were regularly included in daily training practices. Before starting the trials, there was a theoretical explanation and a practical demonstration. For clarity, all abbreviations used in this section of the manuscript have been inserted into Table 1.

### 2.2. Participants

A total of seventeen elite U17 male soccer players (body mass = 69.1 kg ± 5.7; height = 177.7 cm ± 4.7; body mass index = 21.9 ± 2 kg·m^−2^) competing in the Italian Champion-ship volunteered to participate in this study. Data were collected using the GPS-Inertial Measurement Unit (IMU) units Spinitalia v2 (100 Hz) and the EMG system Myontec M-Body 3 (sampling at 1000 Hz and, subsequently, resampled to 100 Hz). Only players with no recent injuries or health problems that could limit their maximum effort were included in the study. The subjects and their legal guardians were informed in detail about all testing and training procedures, and the latter signed a written informed consent form. The local Human Subjects Ethics Committee approved the study in accordance with the Declaration of Helsinki.

### 2.3. Procedures

All tests were conducted on the same training and playing field (home field), with-out changing the grass pitch, and each player was provided with technical clothing suitable for the tests that did not impair their running characteristics. The players first per-formed a series of constant runs to meet the required theoretical standards [36]. Each player completed a maximal sprint of over 30 m following the continuous runs. Each participant executed six trials, one attempt per sprint type. The players were instructed to perform all-out efforts in each trial and could repeat the sprint if unsatisfied with their first attempt. To minimize the effects of fatigue on performance, a five-minute rest period was allowed between trials. Participants were verbally encouraged during the tests to complete each trial as quickly as possible to achieve the best possible results.

*For constant running testing*, four incremental constant running speeds over 50 m were conducted, with required times of ~22.5, ~15, ~11.3, and ~9 s. A US test was then performed, using only the maximum speed reached (as registered by the GPS device) to create an individual profile, as detailed by Grassadonia et al. [36] and explained below (see the *Ad-hoc* Profiling section).

*For the unresisted sprint (US)*, the US protocol involved athletes performing a maximal sprint of over 30 m without any external resistance. The athletes were instructed to sprint with maximal effort from a stationary position, covering the designated distance in the shortest time possible [36,45]. The distance on the field was fixed using metric tape and cones to ensure the perfect visibility for the players (this was maintained for all sprint tests). In the absence of photocells or a radar gun, the sprint performance was determined as the maximal instantaneous velocity recorded by the GPS device during the trial. The US represented the benchmark value for all other sprint conditions for all variables assessed (i.e., muscle activation, MP, EC, and AE).

*For parachute Sprint (PS)*, the PS required athletes to sprint while dragging a parachute (142 × 142 cm parachute size) attached to their waist to ensure the resistance aligned with the body’s center of mass. This added resistance-created drag, challenging the athletes’ ability to maintain speed. The athletes performed maximal sprints over a specified distance of 30 m with the parachute deployed. The PS test is frequently employed to assess an athlete’s ability to generate sprinting under resisted conditions, emphasizing a scenario where athletes encounter external resistance by air [3]. In the present study, we ensured consistency using all players’ identical field positions and parachutes. This approach was crucial for minimizing variables, as air resistance can be unpredictable and difficult to control in field test situations [46]. Nevertheless, during the month of July 2021, the average wind velocity was fairly constant for the time of day of the testing sessions (~16 h 15) across the different days of data collection, ranging from ~17.5 km·h^−1^ to 18.7 km·h^−1^ (data retrieved from weatherspark.com). As for the US, the sprint performance was determined as the maximal instantaneous velocity recorded by the GPS device.

*For the sled sprint (SS)*, in the SS protocol, athletes sprint while towing a sled loaded with various additional loads (i.e., 15 kg, ~22% of body mass [21.86 ± 1.89%]; 30 kg, ~44% of body mass [43.72 ± 3.77%]; 45 kg, ~66% of body mass [65.57 ± 5.66%]; and 60 kg, ~87% of body mass [87.43 ± 7.54%]). The order of the loads used (i.e., SS15, SS30, SS45, and SS60) was randomized for each participant. Despite the well-established limitations of using absolute loads, rather than relative loads [11], the former were utilized due to the limited time available for con-ducting the tests. This approach allowed for more efficient data collection under the given time constraints. The sled was attached via a harness around the trunk and shoulders to distribute the load on the upper body. The athletes performed maximal sprints over the designated distance (i.e., 30 m), with the peak velocity registered by the GPS to be considered for analysis. GPS and EMG data were recorded and analyzed.

*For EMG recording and analysis*, during the experimental trials, the players incorporated textile electrodes into EMG shorts (Myonear Pro, Myontec, Kuopio, Finland). The electrodes, endowed with conductive properties, and their associated wiring were seamlessly integrated into the fabric. These electrodes covered, globally, three major muscle groups on both sides and formed six dif-ferential EMG biosignal channels specifically targeting the quadriceps (Q), hamstrings (H), and glutes (G). The total muscle load, the sum of all EMG signals, was also recorded and considered for the analysis. Two different sizes of shorts (i.e., M and L) were provided, with the participants being individually fitted with the most suitable size. The correct fit of the shorts proved to be a crucial factor, as it allowed for the necessary contact between the electrodes and the skin. This precaution reduced potential movement artifacts during the dynamic activities [47]. Before the participants wore the shorts, a small amount of water was carefully applied to the electrodes to improve signal conduction, a technique recom-mended in previous research [48]. The resulting EMG signals were transmitted and ana-lyzed on a laptop. They were recorded at 1000 Hz and analyzed using Myontec ‘‘Muscle Monitor’’ software version 3.1.0.4 (Kuopio, Finland). The textile electrodes embedded in the shorts were comparable to conventional surface EMG methods in recording lower limb muscle excitation data [47]. Each trial was first processed using a second-order But-terworth bandpass filter (with a bandwidth of 40–200 Hz determined by frequency do-main examination and a signal voltage at a −3 dB cut-off frequency). The filtered signals were then rectified and averaged at 100 Hz. The relative contribution of the Q, H, and G to the whole recorded EMG signals during the US was used as a reference benchmark for all comparisons with the loaded sprints. The EMG system was calibrated before data collection in both static and dynamic conditions to ensure accuracy, as detailed elsewhere [36].

*For GPS and IMU recording and analysis*, integrating EMG data with GPS signals enabled a temporal and kinematic differentiation of the phases. Sprint analyses were performed with personalized spreadsheets. GPS movement patterns were determined based on GPS data (e.g., via di Prampero’s model [34], while integrated GPS-IMU data were used to determine EMG movement patterns (e.g., obtaining the EC_EMG from MP_EMG/Speed_IMU). The GPS devices were calibrated according to the manufacturer’s instructions (Spinitalia S.R.L., Rome, Italy), and the GPS and IMU data were recorded at 50 Hz and 100 Hz, respectively [49]. For data collection, the devices were positioned on the athletes’ lower back, following the procedures explained in previous research [36].

*For ad-hoc profiling*, the profiling was performed before the sprint trials, utilizing an individualized linear profile for each athlete. This profile included slope and intercept and was constructed by plotting the GPS MP and muscle load from all of the EMG signals acquired. A new, simple method was used to recalculate MP and EC from the EMG to ensure accuracy during the profiling (Colli, unpublished), as Grassadonia et al. [21] explained).

*For apparent efficiency (AE)*, AE was calculated from the total work–EC ratio (both expressed in J·kg^−1^·m^−1^) [41]. This was conducted since, to calculate efficiency, the metabolic and mechanical demands must be expressed in the same units. External work was calculated by first using the triaxial integration of acceleration (x, y, z) multiplied by speed to obtain mechanical power. Then, the mechanical power was multiplied and normalized by the time unit (correct condition so that the comparison is not affected by the duration), thus obtaining the external work. Regarding internal work, MP (obtained by EMG) was also used, multiplied, and normalized by the unit of time. Once the internal and external work were obtained, they were summed to obtain the total work. Then, the total work was divided by the net EC. Finally, EC_EMG and MP_EMG were used instead of GPS markers due to their higher accuracy, as demonstrated by Grassadonia et al. [36]. GPS-IMU was only used to estimate external work. A clarifying equation follows:


$$\mathrm{Apparent}\;\mathrm{Efficiency}\;\mathrm{Calculation}\;\mathrm{AE}=\frac{\left[\left(\mathrm{Mechanical}\;\mathrm{Power}\;*\;\mathrm{Time}\;\mathrm{Unit}\right)+\left(\mathrm{Metabolic}\;\mathrm{Power}\;*\;\mathrm{Time}\;\mathrm{Unit}\right)\right]}{\mathrm{Energy}\;\mathrm{Cos}t}$$

### 2.4. Statistical Analysis

Statistical analyses were conducted using the Statistical Package for Social Sciences (SPSS) software, version 25.0 (Chicago, IL, USA), Microsoft Excel 2019 (Redmond, WA, USA), and Statistical Software Jamovi, version 2.3.28. Data are presented as means and standard deviations. In agreement with previously published studies [16,17], the velocity loss percentage (%) and intra-trial differences on a test of GPS and IMU were used to check the speed loss concerning the US. The coefficient of determination (R^2^) was used to assess the goodness of fit of the linear regressions (e.g., in relative muscle contribution and AE). The Kolmogorov-Smirnov test was used to verify if the values followed a normal distribution, while the Wilcoxon signed rank test was used for non-normally distributed data. A 2 × 6 repeated measures ANOVA with determination method (GPS-IMU, EMG) and loading condition (US, PS, SS15, SS30, SS45, and SS60) as factors were used to examine differences in MP and EC. When significant method–load interactions were found, pairwise comparisons with Tukey post-hoc analysis were conducted to confirm all statistical differences. Partial eta squared (η^2^*p*) with threshold values fixed for small (η^2^*p* ≥ 0.01), medium (η^2^*p* ≥ 0.06), and large (η^2^*p* ≥ 0.14) was used to measure the effect size (ES) of the different variables in the ANOVA models and Cohen’s d ES with the corresponding 95% confidence intervals were calculated for pairwise comparisons. Threshold values fixed for Cohen’s d ES were considered small (≥0.2), medium (≥0.5), and large (≥0.8). A significance level of *p* ≤ 0.05 was used for all procedures to determine statistical significance.

## 3. Results

**Figure 1:**
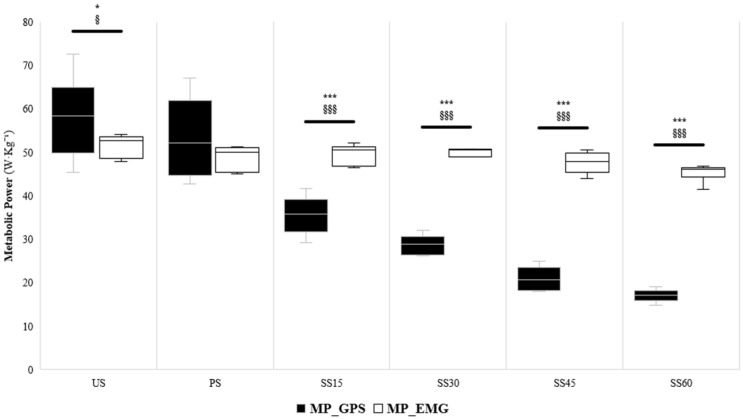
Comparison of the metabolic power determined through the GPS and EMG approaches in the different loading conditions. * *p*-value ≤ 0.05, ** *p* ≤ 0.01, *** *p* ≤ 0.001; § ES ≥ 0.20, §§ ES ≥ 0.50, §§§ ES ≥ 0.80.

Figure 1 displays the comparison of MP determined by GPS and EMG data across the different loading conditions. Significant effects for method (*p* ≤ 0.001; η^2^*p* = 0.53) and load (*p* ≤ 0.001; η^2^*p*= 0.78) were found. When comparing the US with sleds, significant differences were found with the GPS and EMG data (*p* ranging from 0.01 to 0.001 and ES ranging from 0.4 for the US to 1.1–2.1 in SS) but not in the PS (*p* = 0.305). Moreover, a significant method–load interaction was observed (*p* ≤ 0.001, η^2^*p* = 0.78). In the US condition, there were significant differences (Δ11.2%; *p* ≤ 0.01; ES = 0.4 [0.2;0.6]) between MP_GPS and MP_EMG. In the PS condition, MP_GPS and MP_EMG were not significantly different (Δ8.4%; *p* = 0.305; ES = 0.3 [0.1;0.5]). Notably, in the SS15 (Δ−39.6%; *p* ≤ 0.001; ES = 1.1 [0.9;1.4]), SS30 (Δ−73.0%; *p* ≤ 0.001; ES = 1.6 [1.3;1.9]), SS45 (Δ−127.4%; *p* ≤ 0.001; ES = 2.0 [1.6;2.3]) and SS60 (Δ−165.3%; *p* ≤ 0.001; ES = 2.1 [1.7;2.4]) conditions, the MP_GPS values were significantly lower than the MP_EMG.

**Figure 2:**
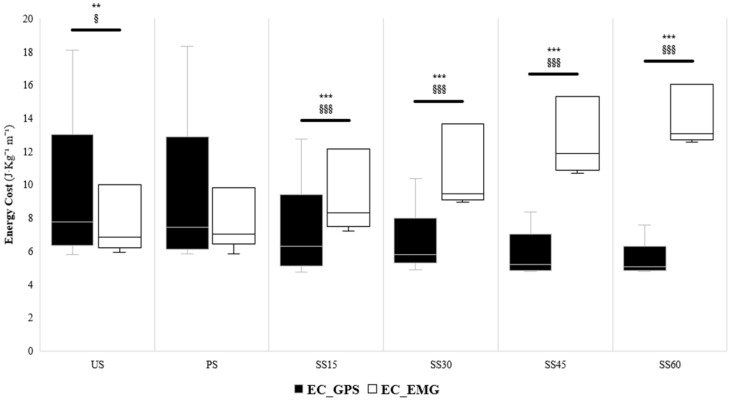
Comparison of the energy cost determined through the GPS and EMG approaches in the different loading conditions. * *p*-value ≤ 0.05, ** *p* ≤ 0.01, *** *p* ≤ 0.001; § ES ≥ 0.20, §§ ES ≥ 0.50, §§§ ES ≥ 0.80.

Figure 2 shows the EC values obtained from both the GPS and EMG approaches in all loading conditions. A significant effect for method (*p* ≤ 0.001; η^2^*p* = 0.57) and load (*p* ≤ 0.001; η^2^*p* = 0.22) was found. When comparing the US with the loaded sprints, significant differences were found with the GPS and EMG methods in all sled conditions (i.e., SS15, SS30, SS45, and SS60, all *p* ≤ 0.001; d ranging from 0.4 with *p* = 0.002 to 0.9–1.8 in sleds) but not in the PS (*p* = 0.102). Moreover, a significant method * load interaction was observed (*p* ≤ 0.001, η^2^*p* = 0.73). In the US condition, significant method–load differences were found between approaches (Δ12.9%; *p* = 0.002; ES = 0.4 [0.2;0.6]). Conversely, no significant differences were identified in the PS between the EC_GPS and the EC_EMG (Δ10.7%; *p* = 0.102; ES = 0.3 [0.1;0.5]). Large significant differences were detected in the SS15 (Δ−38.8%; *p* ≤ 0.001; ES = 0.9 [0.7;1.1]), SS30 (Δ−77.5%; *p* ≤ 0.001; ES = 1.2 [0.9;1.4]), SS45 (Δ−132.4%; *p* ≤ 0.001; ES = 1.4 [1.2;1.7]) and SS60 (Δ−168.46%; *p* ≤ 0.001; ES = 1.8 [1.5;2.1]) conditions, where the EC_GPS was lower than the EC_EMG.

**Figure 3:**
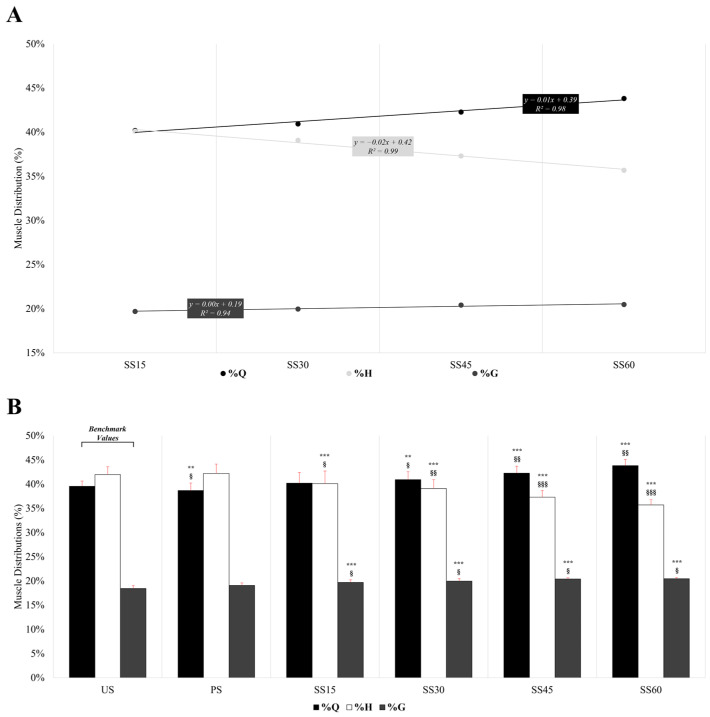
Linear regressions of the relative contribution of the quadriceps, hamstrings, and gluteus muscles as a function of sled load (**A**). Comparison of the muscle distribution in each loading condition (**B**). * *p*-value ≤ 0.05, ** *p* ≤ 0.01, *** *p* ≤ 0.001; § ES ≥ 0.20, §§ ES ≥ 0.50, §§§ ES ≥ 0.80.

**Figure 4:**
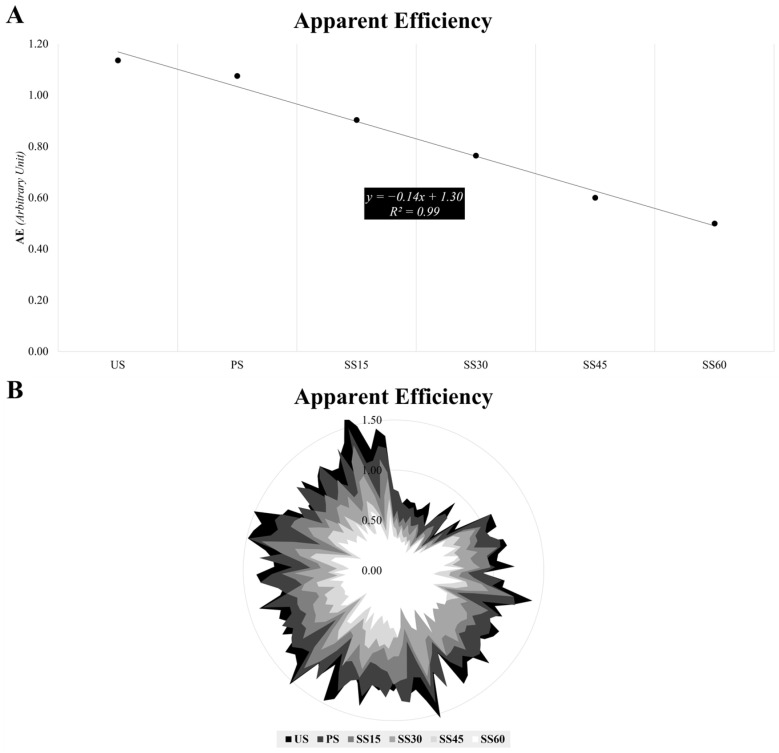
Linear regression of the Apparent Efficiency as a function of sled load (**A**) and qualitative trend states comparison at each 5 m split for a single player (**B**).

Figure 3 depicts the relative muscle contribution of the Q, H, and G (in percentage of the total muscle load detected) in the different sprinting conditions. The muscle’s relative contribution, expressed as a percentage, shows statistically significant differences that grow with increasing overload (Figure 4A,B). A linear increase was observed in the Q and G activation as the load increased (R^2^ = 0.98 and R^2^ = 0.94, respectively). Conversely, a linear decline was found with increasing loads in the H activity (R^2^ = 0.99) (Figure 3A). Figure 3B details the relative contributions of the Q, H, and G in terms of muscle activation. Considering the US and the PS conditions, significant differences were found in the Q (*p* = 0.011; ES = 0.3 [0.1;0.5]) but not in the H (*p* = 0.569; ES = 0.1 [0.1;0.3]) and G (*p* = 0.095; ES = 0.2 [0.0;0.4]). Regarding SS15, no significant changes were found for the Q (*p* = 0.148; ES = 0.1 [0.1;0.3]), but a significantly greater and lower EMG activity was observed for the G (*p* ≤ 0.001; ES = 0.4 [0.2;0.6]) and H (*p* ≤ 0.001; ES = 0.5 [0.3;0.7]), respectively. In the SS30, no significant differences were reported in the relative contribution of the Q (*p* = 0.004; ES = 0.3 [0.1;0.5]), but significantly lower and higher relative muscle activations were registered for the H (*p* ≤ 0.001; ES = 0.7 [0.5;0.9]) and the G (*p* ≤ 0.001; ES = 0.4 [0.2;0.6]), respectively. In the SS45 condition, significantly different muscle activation patterns concerning the US were found for all three muscles assessed, including Q (*p* ≤ 0.001; ES = 0.5 [0.3;0.7]), H (*p* ≤ 0.001; ES = 1.0 [0.8;1.3]), and G (*p* ≤ 0.001; ES = 0.5 [0.2;0.7]). Lastly, for the SS60, the results followed the same trend as in the previous conditions, with significantly higher contributions of the Q (*p* ≤ 0.001; ES = 0.8 [0.6;1.0]) and G (*p* ≤ 0.001; ES = 0.5 [0.3;0.7]) and significantly lower for the H (*p* ≤ 0.001; ES = 1.5 [1.2;1.8]).

Table 2 presents the AE’s descriptive data, and Figure 4 displays the linear regression (R^2^ = 0.99) obtained when plotting AE from the different sprinting conditions. A statistically higher AE_US was found when compared to AE_PS (*p* ≤ 0.001; ES = 0.5 [0.3;0.7]). Likewise, statistically significant differences were found when comparing the former with AE_SS15 (*p* ≤ 0.001; ES = 1.8 [1.5;2.1]), AE_SS30 (*p* ≤ 0.001; ES = 2.4 [2.0;2.7]), AE_SS45 (*p* ≤ 0.001; ES = 2.8 [2.4;3.2]); and AE_SS60 (*p* ≤ 0.001; ES = 3.2 [2.7;3.6]).

## 4. Discussion

Sprinting is essential to many sporting activities, making it crucial to study the muscle engagement and the metabolic and energetic requirements of different sprints [3,11,24,25,50,51,52]. As such, in the current research, alterations in the relative muscle contributions of the Q, H, and G, as well as the EC, MP, and AE, were assessed during resisted sprint efforts using a novel approach recently employed in unloaded sprinting [36]. The main findings were that (1) a greater relative contribution of the Q and G was observed with increasing loads in resisted sprints and, in contrast, lower activations of the H were noted; (2) changes in EC and MP were observed in the sled conditions (i.e., SS15, SS30, SS45 and SS60), with the GPS-derived calculations significantly underestimating these metrics when compared to the EMG-derived values; and (3) an almost perfectly linear decrement in AE was observed when the imposed external resistance increased (R^2^ = 0.99).

Regarding muscle recruitment during resisted sprints, the present findings indicated that PS did not result in different activation patterns of the Q, H, and G compared to the US condition. This outcome was somehow expected, as the parachute introduces only a minimal overload, seemingly insufficient to markedly alter muscle activation. In contrast, when sleds were used, the activation of the Q significantly increased as a function of load as opposed to the H, in which the relative contribution decreased linearly (Figure 3A). Thus, H showed an inverse behavior to Q, which increased and became the primary active muscle as more load was added (due to increased knee flexion and reduced leg stiffness with higher loads [8]). These findings align with those by Zabaloy et al. [8] and van den Tillaar [53] who also reported significantly lower and higher H and Q EMG activity, respectively, with increasing loads in resisted sprinting. For example, in their study, Zabaloy et al. [8] reported decreases in biceps femoris long head activity of ~29% during the acceleration phase (i.e., 0–5 m) and ~17.5% during the maximum velocity phase (i.e., 20–25 m) when comparing US with SS with a load that resulted in a 30% velocity loss in a sample of rugby players. In the Q, and under the same loading conditions, the authors observed increases of ~26% during the acceleration phase and ~41% during the maximum velocity phase in rectus femoris muscle activity [8]. These alterations are partially explained by the acute changes in running mechanics that occur in loaded sprints [8,19] that eventually change muscle recruitment. Therefore, based on the reported disruptions in muscle activity, stiffness, and running kinematics (e.g., reduced stride length, different trunk or lower limb angles) when heavier sled loads are used (e.g., above 30% velocity loss) [8,19], it has been suggested that players may not be “sprinting” but instead, “strength training” [11]. In this regard, SS may presumably have a lower transfer to high-speed actions (e.g., maximum velocity phase of sprinting) being, instead, better suit-ed to improve acceleration capabilities [11,19,20,54,55]. Altogether, the present findings indicate that PS appears to cause fewer changes in muscle activation patterns compared to SS in youth soccer players. Using the former may be advantageous when the goal is to replicate US conditions with a slight overload. However, practitioners should be aware that, with PS, controlling the training load is challenging, more complex, and less controllable (e.g., depends on air resistance and environmental conditions), especially during acceleration as opposed to the maximum velocity phase [3]. Further research is needed to confirm these findings and to fully understand the mechanisms behind the broader effects of PS and SS.

Notably, when sleds were used (i.e., SS15, SS30, SS45, and SS60), the MP derived from the EMG differed significantly from that derived from the GPS, which was not the case in the PS condition. As the load on the sled increased, the GPS data produced a larger error in estimating MP due to GPS calculations being based on acceleration data, which may not provide an estimate of effective energy input in these conditions [32,56,57,58,59]. The present findings indicated that the MP derived from the EMG remained practically constant while the GPS-derived calculation decreased. Thus, with increasing loads, a greater divergence of the data obtained from GPS and EMG was noted (e.g., Figure 1). This may be explained by the fact that MP is the product of EC and speed, and, as EC increases with sled load (Figure 2), but the speed decreases, the MP remains stable when derived from EMG (despite being attained in entirely different ways). This indicates that the MP derived from the external load data (as GPS) might underestimate the effort during the loaded sprint. Therefore, researchers and physical trainers should be cautious and aware when prescribing sprint runs with specialized modalities (e.g., sleds) and use the MP and EC obtained from EMG (rather than GPS-IMU) to assess its effective neuromuscular and energetic commitment. Another aspect worth highlighting is that the variability among GPS splits was greater than that of the EMG, which is essential from a qualitative perspective [36]. Thus, when evaluating actions against resistance, using EMG to determine the actual neuromuscular and metabolic demands properly would be desirable.

Finally, regarding AE, a linear decay (R^2^ = 0.99) was observed with the addition of loads during the sprints (Figure 4A,B), which is a clear indication of the effects of the external resistance on efficiency. In a previous study, Monte et al. [41] analyzed AE during dynamic efforts, such as constant running at different speeds, and concluded that the “apparent” running efficiency increased with the increasing constant speed (10, 13, and 16 km·h^−1^). However, direct comparisons with the present findings are impossible to draw since, to date, no study has investigated AE in resisted sprinting (to the authors’ knowledge). The results of Zabaloy et al. [8] indicate that, as the load increased, the ability to store and release elastic energy (hence, to “recycle” mechanical work) was significantly compromised. The authors noted significantly lower leg stiffness values when external resistance increased, which suggested that stretch-shortening cycle function was impaired under heavier sled load conditions. For all of the above, determining AE could prove to be very useful in practice, as it provides an appropriate response to the effect of different ex-ternal resistance during sprints. From an applied perspective, practitioners should keep in mind that, if the goal is to develop maximum speed capabilities (with short, reactive, and forceful ground contacts where the optimal utilization of the stretch-shortening cycle is key) [28,60,61,62], sled towing should be used with caution [8].

This study has limitations that must be considered when interpreting the results. In particular, the mechanical-energetic engagement [63,64,65,66,67] was evaluated against the same absolute loads which does not account for the variability in athletes’ anthropometric characteristics or performance (i.e., velocity and force production) [1,6,11,52]. However, this decision was made in conjunction with the team’s coaching staff since it was the most time-efficient method to implement in this study during the preseason (despite acknowledging that using load thresholds relative to the athletes’ body mass or speed loss could have improved the study’s accuracy and allowed for further comparison of the data among individuals). The choice between absolute and relative loads significantly influences the interpretation of the results and their real-world applicability. While absolute loads simplify data collection and improve time efficiency, future research using loads relative to body mass or based on velocity loss is necessary to enhance the accuracy and comparability of studies. This approach will ultimately lead to more effective training protocols and outcomes. Also, even though the average wind velocity was fairly constant across the different days of data collection, it is essential to recognize that slight variations in climatic conditions could have influenced PS performance. Finally, the methodological approach used to obtain the AE data limits the comparison with the few previous studies that have analyzed this variable [33,37,38,39,40,41,68]. Further research is needed to determine if and how training adjustments can influence the current results. Nevertheless, the present research represents the first attempt in the literature to capture these data on resisted sprinting (e.g., EC, MP, and AE) and offers a new perspective on sprint evaluation.

## 5. Conclusions

The present findings may help strength and conditioning coaches plan their training sessions more effectively. First, the current results support that muscle activation pat-terns change as a function of load in sprinting. Thus, coaches should be aware that, as load increases, so does the activation of the Q and G as opposed to the H, which become less determinant under heavier loading. Second, practitioners must acknowledge that PS may be preferred when the goal is to replicate US conditions with a slight overload, with-out major disruptions in running mechanics and alterations in muscle involvement. Third, the current data demonstrate that GPS-derived MP and EC underestimate the neuromuscular and metabolic demands imposed on youth soccer players, particularly under heavier load conditions (i.e., >SS15). Therefore, EMG-derived calculations are recommended to better understand the EC and MP of loaded sprinting actions. Finally, coaches and physical trainers should consider the decreased AE and increased EC associated with augmented loads. Future research should investigate parameters such as muscle load and relative recruitment of each muscle group detected, as well as speed, MP, and EC analysis when applying different training methods. These modalities of sprint development, including US, PS, and SS protocols, provide valuable insight into an athlete’s sprinting abilities under various conditions and provide essential data for optimizing training programs and improving performance.

## Figures and Tables

**Table 1:** List of abbreviations.

	Abbreviations
**AE**	Apparent Efficiency
**EC**	Energy Cost
**EMG**	Electromyography
**G**	Gluteus
**GPS**	Global Positioning System
**H**	Hamstrings
**IMU**	Inertial Measurement Unit
**MP**	Metabolic Power
**PS**	Parachute Sprint
**Q**	Quadriceps
**SS**	Sled Sprint
**SS15**	Sled Sprint with 15 kg of additional mass
**SS30**	Sled Sprint with 30 kg of additional mass
**SS45**	Sled Sprint with 45 kg of additional mass
**SS60**	Sled Sprint with 60 kg of additional mass
**US**	Unresisted Sprint

**Table 2:** Descriptive data on the Apparent Efficiency in the different sprinting conditions.

	Apparent Efficiency *(Arbitrary Unit)*
**US**	1.14 ± 0.24
**PS**	1.08 ± 0.23
**SS15**	0.90 ± 0.22
**SS30**	0.76 ± 0.19
**SS45**	0.60 ± 0.14
**SS60**	0.50 ± 0.12

## Data Availability

Data are contained within the article.
